# Innate B cells: oxymoron or validated concept?

**DOI:** 10.12688/f1000research.1-8.v1

**Published:** 2012-08-02

**Authors:** Carl F Ware, Chris Benedict

**Affiliations:** 1 Laboratory of Molecular Immunology, Infectious and Inflammatory Diseases Center, Sanford-Burnham Medical Research Institute, California, 92037, USA; 2 Division of Immune Regulation, La Jolla Institute for Allergy and Immunology, California, 92037, USA

## Abstract

B lymphocytes promote the initial innate interferon response to viral pathogens without the need for antigen receptor activation. B cell dependent IFN production requires the cytokine, lymphotoxin-β. The LTβ pathway is well known to regulate lymphoid organogenesis and homeostasis by differentiating stromal cells and macrophages. However, in response to viral pathogens these same B cell-regulated populations rapidly produce type 1 interferons. Thus, B cells act as innate effector cells via LTβ homeostatic pathways, which serve as innate host barriers to viral pathogens.

## Lymphotoxin-β pathway and innate B cells

The B cell is an icon of the adaptive immune system, secreting a specific antibody that prevents re-infection by pathogens. Although some B cell subsets (e.g., B1 cells) show characteristics of "innate" cells (defined here as cells that do not utilize antibody or antigen receptor genes), the concept of an "innate B cell" somehow doesn't register. However, accumulating evidence validates another view of B cells, one as an innate effector cell initiating the earliest response against viral pathogens, independently of antibody. In two studies, B cells were shown to control the initial type 1 IFN response to very different viral pathogens in lymphoid tissues, cytomegalovirus (CMV, a β-herpesvirus with a large DNA based genome) ^1^ and vesicular stomatitis virus (VSV, a small RNA virus that causes lympho-neurotropic pathogenicity) ^2^.

Schneider *et al.* ^1^ established the B cell dependence of the IFNβ response to infection with CMV. This innate IFN defense mechanism was surprisingly independent of Toll-like receptor pathways, but required the Lymphotoxin (LT)-β receptor signaling pathway, part of the larger superfamily of cytokines related to TNF ^3, 4^. Conditional deletion of the LTβ gene in B cells, but not T cells, provided the key evidence pinpointing the involvement of LTβ in B cells in the initial IFN response to CMV. The LT-IFN response occurs rapidly, initiating within a couple of hours after infection, well before adaptive immunity could contribute. Expression of the IFNβ gene occurred primarily in virus-infected stromal cells in the spleen and accounted for the majority of the circulating IFNαβ. Blocking the LT-IFN pathway resulted in destruction of the splenic architecture and an apoptotic collapse of T and B lymphocytes ^5^.

Moseman *et al.* ^2^ demonstrated the critical role of the B cell dependent LT-IFN defense pathway in response to VSV. Importantly, antibody deficient μMT and DHLMP2a mice revealed IFNβ expression in response to VSV occurred independently of B cell antigen receptor. In the absence of LTβ or IFN signaling, VSV infected the lymphatic neurons and spread into the central nervous system with ensuing paralysis. These results provide strong evidence for the innate action of B cells through the LT-IFN pathway. The effectiveness of the LT-IFN pathway against two distinct pathogens suggests a more generalized role in host defense.

## The architecture of host defense

**Figure 1.**
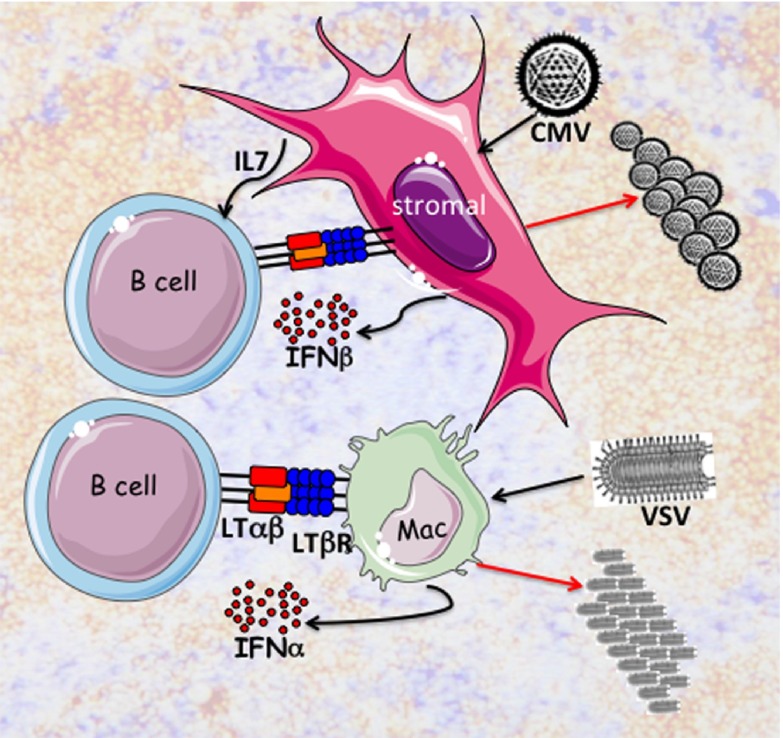
Innate B cells initiate production of type 1 interferons (IFNαβ). B cells express the TNF-related ligand LTαβ that specifically engages the LTβR expressed in lymphoid tissue stromal cells (pink) and myeloid lineage cells including subcapsular macrophages (green). The B cell to stromal cell interaction maintains the homeostasis of lymphoid tissues through secretion of chemokines and IL7, which enhance the expression of LTαβ. Cytomegalovirus (CMV) infects stromal cells (ERTR7+ fibrocytes) in the splenic marginal zone from which IFNβ is rapidly expressed and secreted. B cell expression of LTαβ is also required for CD169+ subcapsular macrophages in lymph nodes. Vesicular stomatitis virus (VSV) infects subcapsular macrophages inducing production of IFNα. Virus replication and progeny are produced (red arrows) in the permissive stromal cells or CD169+ macrophages. IFNαβ protect uninfected cells in the surrounding microenvironment.

The current evidence indicates the innate B cell driven LT-IFN pathway operates during infections in lymphoid tissues. A convergence of recent results may explain this observation. Mouse CMV productively infects reticular fibroblasts in the splenic marginal zone, but also subcapsular sinus macrophages in lymph nodes that express high CD169+ (SIGLEC1) (Figure 1) ^6^. Interestingly, CD169+ macrophages uniquely support VSV infection in lymph nodes and provide the primary source of IFNα during the initial phase of infection with VSV ^7^. Importantly, these CD169+ macrophages require the LTαβ-LTβR pathway to populate the subcapsular regions in lymph nodes and the marginal sinus of the spleen ^8, 9^. LTβ receptor signaling regulates stromal cell expression of homeostatic chemokines (e.g., CXCL13, CCL21) that help to position CD169+ macrophages in lymph nodes and spleen. In the absence of LTβR signaling, CD169+ subcapsular macrophages no longer reside in lymph nodes, depriving the virus of a permissive cell for replication, with a commensurate loss in IFNα production. The mechanism underlying the permissiveness of the CD169+ macrophages is not entirely understood, however Khanna and Lefrancois ^10^ point out that these macrophages have limited capacity to respond to IFN due to expression of *Usp18* ^11^ encoding an ISG15-deconjugating peptidase that destabilizes multiple antiviral proteins induced by IFN ^12^.

Lymphoid organs provide the structural environment that positions key cells, such as the sinus lining macrophages, directly in the flow of lymph and blood in order to capture pathogens ^13^. Yet, intentionally providing a pathogen with a source of permissive cells seems counterintuitive as a defense strategy. However, amplifying the level of viral antigens to increase presentation to adaptive immune cells could counterbalance this potential danger. Moreover, the powerful selective pressure that the IFN system places on the pathogen is relieved in the permissive macrophage, potentially limiting the emergence of mutant viruses resistant to IFN. Neighboring cells that remain responsive to IFN signaling should be protected, corralling the pathogen within this macrophage-populated niche.

The Lymphotoxin-β pathway orchestrates the embryonic development of lymph nodes and Peyer’s Patches ^14– 16^. In the adult, B lymphocytes constitutively expressing LTβ are the primary cells responsible for the maintenance of the microarchitecture of the spleen and lymph nodes. Thus, this LTβ-dependent developmental pathway is reflected in the adult as an innate B cell host defense mechanism. Recent evidence indicates that bacterial pathogens are controlled in part by innate acting B cells utilizing pattern-recognition receptors and producing granulocyte-macrophage colony-stimulating factor ^17^.

Together these findings validate the notion that adaptive immune cells can mediate effector functions independent of antigen receptor activation thus serving as innate effectors. Conversely, innate effector cells, NK cells display immunologic memory ^18^, an iconic trait of adaptive immunity. These observations indicate the conventional notion of innate and adaptive cells is in need of revision.
