# Supplementary material for: Innate B cells: oxymoron or validated concept?
Source: F1000Res. 2012 Aug 2;1:8. [Version 1] doi: 10.12688/f1000research.1-8.v1 (PMC3751138; doi:10.12688/f1000research.1-8.v1)
Supplement: Peer review history

# Peer review: Referee response for version 1

**Published:** 2012-08-15

**Authors:** Mariapia Delgi-Esposti

**Affiliations:** Experimental Immunology, The University of Western Australia, WA 6009, Australia

I have read this submission. I believe that I have an appropriate level of expertise to confirm that it is of an acceptable scientific standard.

# Peer review: Referee response for version 1

**Published:** 2012-08-14

**Authors:** Peter Ghazal

**Affiliations:** University of Edinburgh Medical School, Edinburgh, EH16 4SB, UK

This commentary summarises recent findings which point to a previously under-appreciated role of B-cells acting as innate effector cells in immunity.

The authors build on the notion for a continuum of activity between the innate and adaptive arms of the immune system, integrated at the cellular level via B-cells. Accordingly, this commentary convincingly argues for reconsideration of the conventional view of the role played by innate and adaptive cells of the immune system.

I have read this submission. I believe that I have an appropriate level of expertise to confirm that it is of an acceptable scientific standard.

# Peer review: Referee response for version 1

**Published:** 2012-08-06

**Authors:** Barry Rouse

**Affiliations:** Department of Pathobiology, College of Veterinary Medicine, The University of Tennessee, Knoxville, TN, USA

This succinct scholarly review has an interesting story but its title could mislead.

Accordingly it gives a nice account about how B cells subserve a protective function other than by their main raison d’etre (antibody production). What it does not do is to provide any evidence for contradictory terminology. Thus the primary function of B cells remains adaptive immunity with their foray into innate immunity also possible, just like countless other cell types. Cells, like us, have many activities some of which are primary and pay the bills and others minor distractions- like surfing if you happen to live in San Diego!

I have read this submission. I believe that I have an appropriate level of expertise to confirm that it is of an acceptable scientific standard, however I have significant reservations, as outlined above.
